# Validation of the Japanese version of the patterns of activity measure-pain in individuals with chronic pain

**DOI:** 10.1186/s13030-022-00248-z

**Published:** 2022-09-04

**Authors:** Kiyoka Enomoto, Tomonori Adachi, Akira Mibu, Katsuyoshi Tanaka, Sei Fukui, Miho Nakanishi, Narihito Iwashita, Jun Sasaki, Tomohiko Nishigami

**Affiliations:** 1 Graduate School of Human Sciences, Osaka University, Osaka, Japan; 2 Japan Society for the Promotion of Science, Tokyo, Japan; 3 Graduate School of Human Development and Environment, Kobe University, Hyogo, Japan; 4 Pain Management Clinic, Shiga University of Medical Science Hospital, Shiga, Japan; 5 Department of Physical Therapy, Konan Women’s University, Hyogo, Japan; 6 Department of Physical Therapy, School of Health Science, Bukkyo University, Kyoto, Japan; 7 Department of Anesthesiology, Shiga University of Medical Science, Shiga, Japan; 8 Department of Physical Therapy, Faculty of Health and Welfare, Prefectural University of Hiroshima, 1-1, Gakuen-chou, Mihara, Hiroshima, 723-0053 Japan

**Keywords:** Chronic pain, Activity pattern, Avoidance, Overdoing, Pacing

## Abstract

**Background:**

The Patterns of Activity Measure-Pain (POAM-P) is a self-report questionnaire that measures avoidance, overdoing, and pacing in individuals with chronic pain. We aimed to develop and confirm the psychometric properties of the Japanese version of the POAM-P(POAM-P-J) in Japanese individuals with chronic pain.

**Methods:**

We recruited 147 Japanese individuals with chronic pain (106 women; mean age 64.89 ± 12.13 years). The individuals completed the POAM-P-J, the Brief Pain Inventory (BPI), and the Hospital Anxiety and Depression Scale (HADS). The following psychometric properties of the POAM-P-J were confirmed: structural validity, internal consistency, test–retest reliability, and concurrent validity.

**Results:**

We tested factor structure via confirmatory factor analyses (CFA). We chose the 3-factor model with six covariances. The POAM-P-J’s internal consistency and test–retest reliability were acceptable to good (*α* = 0.79–0.86; ICC = 0.72–0.87). The avoidance and overdoing subscales were positively associated with pain severity, pain interference, and anxiety measures (all *p* < 0.05), but the pacing subscale was not significantly associated with these pain-related measures.

**Conclusions:**

Although the structural validity of the POAM-P-J remains questionable, its internal consistency, test–retest reliability, and concurrent validity were confirmed. The POAM-P-J is useful in both research and clinical practice for evaluating the activity patterns of Japanese patients with chronic pain.

## Background

The prevalence of chronic pain ranges from 15.4% to 39.3% in Japan [1, 2]. Chronic pain frequently interferes with physical activities. Studies have shown that individuals with chronic pain are less or no longer able to participate in various activities such as employment, housework, or social activities [3].

Individuals who suffer from chronic pain are known to present three characteristic activity patterns: avoidance, overdoing, and pacing [4]. Avoidance means to escape from or avoid pain-associated activities. According to the fear-avoidance model, pain catastrophizing causes an individual to fear and avoid certain activities, resulting in lower levels of activity [5]. Meanwhile, overdoing (also called “persistence” [6], “endurance” [7], or “confronting” [8]) is defined as the tendency to continue with activities despite pain. While it seems beneficial for the short term, it eventually leads to overuse and increases pain and disability [7]. Finally, pacing is characterized by breaking tasks into smaller pieces, taking frequent short rests, and slowing down [9, 10]. From a theoretical perspective, avoidance and overdoing are considered maladaptive whereas pacing is regarded as an adaptive strategy for chronic pain management [4, 11].

To assess the activity patterns of individuals with chronic pain, several self-report questionnaires have been developed. Some widely known instruments are the Patterns of Activity Measure-Pain (POAM-P) [4], the Pain and Activity Relations Questionnaire (PARQ) [8], the Avoidance–Endurance Questionnaire (AEQ) [12], and the Chronic Pain Coping Inventory (CPCI) [13]. Among them, the POAM-P is useful as it can simultaneously measure avoidance, overdoing, and pacing [4]. The original version consists of a 3-factor subscale (avoidance, overdoing, and pacing), with each subscale containing 10 items [4]. Although the POAM-P has already been translated into French [14], Spanish [15], Dutch [16], and Turkish [17], a Japanese version has not been developed.

Previous studies have shown that the relations between the three activity patterns of the POAM-P and pain-related outcomes are different from the theoretical perspective. The POAM-P’s avoidance subscale is consistently associated with more pain, disability, and psychological distress [4, 6, 14, 16–20]. Meanwhile, the relation between the POAM-P’s overdoing subscale and pain outcomes is controversial; while some studies have reported an association between overdoing subscale and more disability and psychological distress [4, 18], some have found that overdoing subscale was less associated [14, 19] or not entirely associated with these outcomes [6, 16, 20]. Pacing subscale has also been reported as having conflicting results regarding the relationship between pain outcomes. Some studies found that pacing subscale was associated with less disability and psychological distress [4, 20], while other studies found that pacing subscale was associated with more pain, disability, and psychological distress [6, 16].

This study sought to confirm the reliability and validity of the Japanese version of the POAM-P (POAM-P-J). For concurrent validity, we based our hypothesis on the theoretical background of activity patterns [4]. Hence, avoidance and overdoing subscales would be associated with more pain, disability, and psychological distress. On the other hand, pacing subscale would be linked to less pain, disability, and psychological distress.

## Methods

### Translating the POAM-P into Japanese

The translation and cross-cultural adaptation process followed the guidelines [21]. First, three individuals (two psychologists and one physical therapist) translated the original POAM-P into Japanese. The three resulting Japanese versions of the POAM-P were then examined by two psychologists (K. E. and T. A.) and the corresponding author (T. N.), who discussed any differences in the contents of the translated items and resolved them via consensus. Thus, the first consensual version of the POAM-P-J was completed. Second, this version of the POAM-P-J was back translated from Japanese into English by a native English speaker, and the output was checked and approved by the developer of the original POAM-P. This was the second consensual version of the POAM-P-J. Third, we conducted a pretest involving five patients with chronic pain (3 women; mean age was 54.6), who answered the second version of the POAM-P-J to verify that the items can be clearly understood. However, since some of the patients mentioned that they could not comprehend the meaning of “activity,” we decided to add a sentence that provided an example of it. The developer of the POAM-P then approved the addition of this sentence and gave some other examples of “activity,” such as washing the dishes, going for a walk, preparing a meal, doing paperwork at your desk, watching a movie, reading a book, and others. Finally, the definitive version of the POAM-P-J was completed [22].

### Participants

We recruited participants from four medical facilities: one pain clinic at a university hospital, two general-hospital rehabilitation units, and one neurosurgery department at a clinic. The inclusion criteria were (1) a history of pain lasting three months or more and (2) age 20–80. The exclusion criteria were (1) organic diseases that affect behavior such as fracture, malignancy, or inflammation and (2) pregnancy or the possibility of pregnancy.

### Measures

#### Demographic variables

The participants provided the following information regarding their backgrounds: age, sex, pain duration, pain location, education level, marital status, and employment status. For pain location, multiple choices were allowed.

#### Activity patterns

The POAM-P is a 30-item self-report questionnaire that measures three activity patterns in patients with chronic pain: avoidance, overdoing, and pacing [4]. Each subscale contains 10 items. Respondents rated each item describing how they usually perform their daily life activities by rating each item on a 5-point scale (0 = not at all to 4 = all the time). The scores for each subscale range from 0 to 40, and higher scores indicate a higher degree of avoidance, overdoing, or pacing. The original POAM-P has good internal consistency; the Cronbach’s *α* coefficients of its scales were 0.86 for avoidance, 0.90 for overdoing, and 0.94 for pacing [4].

#### Pain severity and interference

The Brief Pain Inventory (BPI) consists of two domains: pain severity and pain interference [23, 24]. For pain severity, which includes four items, the participants were asked to rate their pain severity in the past 24 h as “worst,” “least,” or “average” and their “current” pain severity from 0 (no pain) to 10 (pain as severe as you can imagine). We analyzed the average score of the four items. Meanwhile, pain interference includes seven items that assess the extent to which pain has interfered with seven of the participants’ daily activities (general activity, mood, walking ability, normal work, relations with other people, sleep, and enjoyment of life). Participants scored these using a numerical scale (0 = does not interfere and 10 = completely interferes). The pain interference score was calculated by averaging the ratings of the seven items. In the current sample, the Cronbach’s *α* coefficients were 0.86 for pain severity and 0.91 for pain interference.

#### Anxiety and depression

The Hospital Anxiety and Depression Scale (HADS) consists of 14 items in two subscales: anxiety (HADS-A) and depression (HADS-D) [25, 26]. The two subscales include seven items rated on a 4-point scale. These subscales can range from 0 to 21, with higher scores indicating a greater degree of anxiety or depression. In the current sample, the Cronbach’s *α* coefficients were 0.74 for the HADS-A and 0.79 for the HADS-D.

### Procedure

The participants were asked to complete the questionnaires during the waiting time in their outpatient visits. To confirm test–retest reliability, those who revisited the medical facilities within 50 days were asked to retake the POAM-P-J. The data were collected from August 2017 to May 2019. All participants provided written informed consent, and the Institutional Ethics Committee of Konan Women’s University approved the study protocol (No: 2016011; Admission date: May 22, 2017).

### Statistical analysis

We used R software (version 3.6.1) for all statistical analyses [27]. At least 5 to 10 participants per item of the questionnaire should be needed to conduct confirmatory factor analysis [28]. Therefore, we targeted 150 participants for recruitment in this study.

First, we calculated the participants’ demographic characteristics using descriptive statistics. Then, we evaluated the structural validity of the POAM-P-J by performing the confirmatory factor analysis (CFA) using the package “lavaan” [29]. To assess factor structure, we used the following fit indices: chi-square goodness-of-fit index (χ^2^: a nonsignificant result at a 0.05 threshold indicates good fit [30]), normed chi-square (χ^2^/*df*: a value below 2 indicates an acceptable fit [30]), root mean square error of approximation (RMSEA: RMSEA < 0.08 indicates adequate fit [31]), standardized root mean square residual (SRMR: SRMR < 0.08 indicates acceptable fit [30]), comparative fit index (CFI: CFI > 0.95 indicates good fit [31]), and the Akaike information criterion (AIC: a smaller value indicates a more parsimonious model fit [31]).

We then evaluated internal consistency and test–retest reliability using Cronbach’s alpha and intraclass correlation (ICC) coefficients, respectively. Cronbach’s α coefficient > 0.70 indicates sufficient internal consistency [32], and ICC coefficient > 0.75 indicates good test–retest reliability [33]. We used Pearson correlation coefficients to assess the interscale correlations of the POAM-P-J. To determine concurrent validity, we calculated the Pearson correlation coefficients between the POAM-P-J and the other measures.

In this study, we set the statistical significance at *p* < 0.05. There were a few missing values for all study measures (0.2%). In the case of missing variables, we applied the full-information maximum-likelihood method for CFA and the pairwise approach for Pearson correlation analysis.

## Results

### Demographic characteristics of the participants

A total of 151 participants completed the questionnaires, four of whom were excluded for the following reasons: one did not provide their age, two provided incomplete pain duration information, and one did not complete the HADS questionnaire. Therefore, data obtained from the remaining 147 participants were analyzed.

Table 1 summarizes the demographic characteristics and mean values of the study measures. Out of the total number of participants, 106 (72.1%) were women, and their mean age was 64.89 (*SD* = 12.13). Most of them received at least a high school education (84.4%) and were married (72.8%) while 30.6% were employed. Their average pain duration was 81.00 months (*SD* = 75.08), with lower limbs as the most frequently reported pain location (69.4%). Twenty-nine participants (19.7%) experienced pain in more than three areas. Eighteen participants (12.2%) answered the POAM-P-J twice to assess test–retest reliability. The average number of days between the two-time points was 21.06 ± 10.64.

**Table 1:** Demographic characteristics and mean values of measures

Variables		Mean (SD)	n (%)
Age (years)		64.89 (12.13)	
Sex	Men		41 (27.9%)
	Women		106 (72.1%)
Pain duration (months)		81.00 (75.08)	
Pain location	Head, face, or mouth		14 (9.5%)
	Cervical		27 (18.4%)
	Upper shoulder or upper limbs		44 (29.9%)
	Thoracic		7 (4.8%)
	Abdominal		4 (2.7%)
	Low back		52 (35.4%)
	Lower limbs		102 (69.4%)
	Pelvic		7 (4.8%)
	Anal, perineal, or genital		3 (2.0%)
	More than three locations		29 (19.7%)
Education level	Junior high		23 (15.6%)
	High		83 (56.5%)
	Vocational		18 (12.2%)
	Junior college		12 (8.2%)
	Undergraduate		9 (6.1%)
	Graduate school		2 (1.4%)
Marital status	Married		107 (72.8%)
	Unmarried		12 (8.2%)
	Divorced		10 (6.8%)
	Widowed		17 (11.6%)
	No response		1 (0.7%)
Employment status	Full-time employment		28 (19.0%)
	Part-time employment		17 (11.6%)
	Student		1 (0.7%)
	Homemaker		37 (25.2%)
	Retirement		12 (8.2%)
	Suspension or retirement due to pain		19 (12.9%)
	Not working unrelated to pain		18 (12.2%)
	Others		15 (10.2%)
Pain Severity (BPI)		4.16 (1.78)	
Pain Interference (BPI)		4.19 (2.35)	
Anxiety (HADS-A)		6.30 (3.52)	
Depression (HADS-D)		6.47 (3.98)	

*Abbreviations*: *BPI* Brief Pain Inventory, *HADS* Hospital Anxiety and Depression Scale

### Structural validity of the POAM-P

**Fig. 1:**
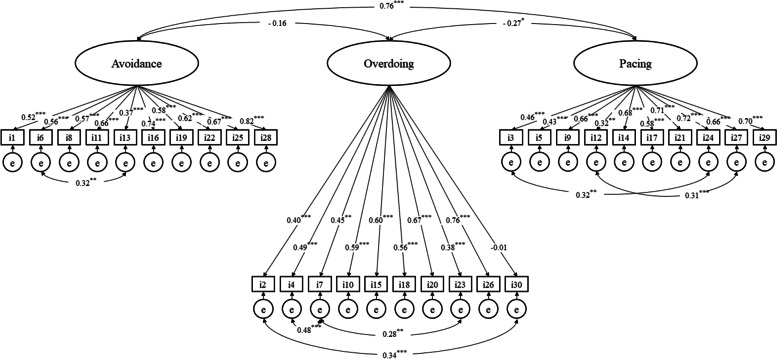
The three-factor model of the Japanese version of the Patterns of Activity Measure-Pain. ^***^p < 0.001. Note: “i” represents the item and “e” represents error. We reported standardized parameter estimate values

We tested the three-factor model of the original POAM-P via CFA: avoidance subscale (items 1, 6, 8, 11, 13, 16, 19, 22, 25, and 28), overdoing subscale (items 2, 4, 7, 10, 15, 18, 20, 23, 26, and 30), and pacing subscale (items 3, 5, 9, 12, 14, 17, 21, 24, 27, and 29). The first model failed to indicate sufficient fit (*χ*^2^(402) = 825.303, *p* < 0.001, *χ*^2^/*df* = 2.053, RMSEA = 0.088, SRMR = 0.130, CFI = 0.728, and AIC = 12,294.564; Table 2). To improve this model, we allowed six error covariances between items with modification indices above 10. Such error covariances were selected due to the overlap of meaning. In the second model, although *χ*^2^, SRMR, and CFI showed poor fit, *χ*^2^/*df* and RMSEA indicated adequate fit (*χ*^2^(396) = 724.021, *p* < 0.001, *χ*^2^/*df* = 1.828, RMSEA = 0.078, SRMR = 0.129, CFI = 0.789, and AIC = 12,204.542; Table 2). This model was selected to retain compatibility with the original version. Figure 1 presents the factor loadings of POAM-P-J.

**Table 2:** Fit indices of the POAM-P-J

	χ^2^ (*df*)	*p*	χ^2^/ *df*	RMSEA (90%CI)	SRMR	CFI	AIC
Model 1: 3-factor model	825.303 (402)	< 0.001	2.053	0.088 (0.079–0.096)	0.130	0.728	12,294.564
Model 2: 3-factor model with 6 covariances	724.021 (396)	< 0.001	1.828	0.078 (0.069–0.087)	0.129	0.789	12,204.542

*Abbreviations*: *POAM-P-J* Japanese version of the Patterns of Activity Measure-Pain, *RMSEA* Root mean square error of approximation, *CI* Confidence interval, *SRMR* Standardized root mean square residual, *CFI* Comparative fit index, *AIC* Akaike information criterion

### Reliability and interscale correlations of the POAM-P-J

Table 3 shows the mean scores, reliabilities, and interscale correlations of the scales. The Cronbach’s alpha coefficients for the POAM-P-J scales ranged from 0.79 to 0.86. The ICC ranged from 0.72 (95% CI: 0.40–0.89) to 0.87 (95% CI: 0.66–0.95).

**Table 3:** Reliability and interscale correlations of the POAM-P-J

POAM-P-J scale	Mean	*SD*	*α* (95% CI)	*ICC* (95% CI)	Overdoing	Pacing
Avoidance	21.31	8.63	0.86 (0.83 to 0.89)	0.73 (0.39 to 0.89) ^***^	0.12 (− 0.04 to 0.28)	0.63 (0.51 to 0.72) ^***^
Overdoing	18.50	7.00	0.79 (0.74 to 0.84)	0.72 (0.40 to 0.89) ^***^	―	0.07 (− 0.09 to 0.24) ***
Pacing	23.80	7.78	0.85 (0.81 to 0.88)	0.87 (0.66 to 0.95) ^***^		―

*Abbreviations*: *POAM-P-J* Japanese version of the Patterns of Activity Measure-Pain, *CI* Confidence interval, *ICC* Intraclass correlation, *SD* Standard deviation

^***^ *p* < 0.001

The interscale correlations showed that avoidance subscale was moderately correlated with pacing subscale (*r* = 0.63, *p* < 0.001) whereas overdoing subscale was not significantly correlated with avoidance and pacing subscales.

### Concurrent validity of the POAM-P-J

Table 4 shows the correlations between the POAM-P-J scales and the study measures. As expected, avoidance subscale had significant weak positive correlations with measures of pain severity (*r* = 0.19, *p* < 0.05), pain interference (*r* = 0.28, *p* < 0.001), anxiety (*r* = 0.17, *p* < 0.05), and depression (*r* = 0.17, *p* < 0.05). Overdoing subscale also had significant weak positive correlations with pain severity (*r* = 0.23, *p* < 0.01), pain interference (*r* = 0.23, *p* < 0.01), and anxiety (*r* = 0.28, *p* < 0.001). Only the depression measure did not have a significant correlation with the overdoing subscale (*r* = 0.15, *n.s.*). By contrast, pacing subscale was not correlated with pain severity, pain interference, anxiety, or depression.

**Table 4:** Correlations between the POAM-P-J subscales and pain severity, pain interference, anxiety, and depression

POAM-P-J scale	Pain Severity (BPI)	Pain Interference (BPI)	Anxiety (HADS)	Depression (HADS)
Avoidance	0.19 (0.03 to 0.35)^*^	0.28 (0.12 to 0.42) ^***^	0.17 (0.00 to 0.32)^*^	0.17 (0.01 to 0.32)^*^
Overdoing	0.23 (0.07 to 0.38)^**^	0.23 (0.07 to 0.38) ^**^	0.28 (0.12 to 0.43)^***^	0.15 (− 0.02 to 0.30)
Pacing	0.06 (− 0.11 to 0.22)	0.11 (− 0.06 to 0.27)	0.10 (− 0.06 to 0.26)	0.07 (− 0.10 to 0.23)

*Abbreviations*: *POAM-P-J* Japanese version of the Patterns of Activity Measure-Pain, *BPI* Brief Pain Inventory, *HADS* Hospital Anxiety and Depression Scale

^***^ *p* < 0.001, ^**^ *p* < 0.01, ^*^ *p* < 0.05

## Discussion

The present study aimed to examine the psychometric properties of the POAM-P-J. We adopted the 3-factor structure of the POAM-P, but some fit indices showed poor fit to data. The POAM-P-J, meanwhile, showed good internal consistency and test–retest reliability. Overall, avoidance and overdoing subscales were associated with higher pain severity, pain interference, and anxiety whereas pacing subscale was not associated with these outcomes.

The structural validity of the POAM-P-J remains questionable. Item 30 displayed a low factor loading, which is similar to the Turkish version of the POAM-P [17]. In the Turkish version of the POAM-P, item 30 was kept so that the original scale structure was not distorted. We followed the Turkish version of the POAM-P, so item 30 was retained. In the current study, sample bias may have contributed to the poor to acceptable fit of POAM-P-J. Although the samples of the original and Turkish versions of the POAM-P consisted of individuals with chronic primary pain with an average age of 40 years [4, 17], the sample in this study consisted of individuals with pain in the lower limbs with an average age of 60 years. Thus, a possibility exists that the sample may include individuals with knee osteoarthritis, which led to their tendency to display less avoidance or overdoing compared with individuals with chronic primary pain. However, structural validity was only confirmed for the Turkish and Japanese versions of the POAM-P. It is necessary to examine the factor structure in different populations in the future.

The POAM-P-J has shown good reliability values. However, the number of participants who answered the POAM-P-J twice (*N* = 18) was small in the current study. A previous study reported that the POAM-P has good test–retest reliability with a sufficient sample size [14]. Succeeding research efforts would therefore need to examine the test–retest reliability of the POAM-P-J with a sufficient Japanese sample.

Meanwhile, according to interscale correlations, the avoidance subscale was moderately correlated with the pacing subscale in this study, and studies have reported the same magnitude of correlations between the two subscales (*r* = 0.46–0.56) [6, 14, 15, 19]. According to a meta-analysis, some pacing items reflected the content of pain-contingent behaviors, with some overlap between the pacing and avoidance subscales [34]. Thus, the validity of POAM-P-J was confirmed.

Although the present results of confirmatory factor analysis showed a significant correlation between overdoing and pacing, the present results of interscale correlations did not show a significant correlation between these factors. This is because a magnitude of the correlation between latent variables that do not include measurement errors tends to inflate relative to a magnitude of the correlation between observed variables [35].

Concurrent validity results showed a positive association between the avoidance and overdoing subscales and measures of pain-related outcomes. Such an association is the same as that in the original study [4] and is considered to confirm the concurrent validity of the POAM-P-J. However, overdoing subscale was not significantly associated with depression. Some studies have reported that the overdoing subscale was not significantly associated with depression [6, 20]. Hasenbring, who proposed the avoidance–endurance model [7], identified two types of overdoing: one associated with positive affect and the other associated with depressive mood. The overdoing subscale of the POAM-P might include these two types, which can therefore be linked to the lack of association between depression and POAM-P-J’s overdoing subscale.

Contrary to the hypothesis, the pacing subscale was not significantly associated with pain-related measures. It is speculative, but these results have two possible reasons. First, pacing may have adaptive and maladaptive components. Pacing is intended to increase activity levels, conserve energy for important activities, and reduce pain [9, 10, 15], but patients may perceive it as limiting when compared to activity levels prior to pain onset [6]. The pacing of the POAM-P includes adaptive and maladaptive aspects, which might have contributed to the lack of association between the pacing subscale and other pain-related outcomes. Second, sample bias might have affected these results. As mentioned above, the study population included many older individuals. According to previous studies [36], older individuals have lower levels of physical activity than younger people. Older individuals may naturally divide tasks into smaller pieces and take frequent short breaks because of aging. The pacing subscale in our study was not associated with pain-related outcomes, possibly because the participants in this study used activity pacing because of aging rather than because of pain.

This study has several limitations. First, the number of people who answered the POAM-P-J twice was small. Second, we did not know each participant’s diagnosis, as the inclusion criteria included only a pain history of three months or more. Third, the participants’ average age was high, and the proportion of those who suffered from lower-limb pain was high as well. Therefore, sampling bias might have occurred. In this study, unlike previous ones [4, 6, 14, 15, 19, 20], overdoing subscale was not significantly associated with avoidance or pacing subscales. Such sampling bias might have affected the relation between overdoing and the two other activity patterns. Fourth, although the validity and reliability of the POAM-P-J were acceptable, these results were based on a questionable factor structure. Further research is needed to examine the psychometric properties of the POAM-P-J.

## Conclusions

We developed the POAM-P-J and examined its psychometric properties. Although its structural validity is in question, its reliability and concurrent validity were confirmed. POAM-P-J is useful for assessing the activity patterns of Japanese patients with chronic pain for research and clinical practice.

## Data Availability

The datasets used and analyzed during the current study are available from the corresponding author on reasonable request.

## Abbreviations

POAM-P: Patterns of Activity Measure-Pain
POAM-P-J: Japanese version of the patterns of Activity Measure-Pain
BPI: Brief Pain Inventory
HADS: Hospital Anxiety and Depression Scale
CFA: Confirmatory Factor Analysis
ICC: Intraclass correlation
RMSEA: Root Mean Square Error of Approximation
SRMR: Standardized Root Mean square Residual
CFI: Comparative Fit Index
AIC: Akaike Information Criterion
